# Variation in Interleukin 6 Receptor Gene Associates With Risk of Crohn’s Disease and Ulcerative Colitis

**DOI:** 10.1053/j.gastro.2018.05.022

**Published:** 2018-08

**Authors:** Constantinos A. Parisinos, Stylianos Serghiou, Michail Katsoulis, Marc Jonathan George, Riyaz S. Patel, Harry Hemingway, Aroon D. Hingorani

**Affiliations:** 1 Institute of Health Informatics Research, Faculty of Population Health Sciences, University College London, London, UK; 2 Health Research and Policy, Epidemiology, Stanford University, Stanford, CA; 3 Centre for Clinical Pharmacology, Division of Medicine, University College London, London, UK; 4 Institute of Cardiovascular Science, Faculty of Population Health Sciences, University College London, London, UK

**Keywords:** SNP, Genetics, Mendelian Randomization, Drug Target Validation, CD, Crohn’s disease, CI, confidence interval, gp130, glycoprotein 130, IBD, inflammatory bowel disease, IL6, interleukin 6, IL6R, IL6 receptor, MR, Mendelian randomization, OR, odds ratio, s-IL6R, soluble IL6R, RCT, randomized controlled trial, SNP, single nucleotide polymorphism, UC, ulcerative colitis

## Abstract

Interleukin 6 (IL6) is an inflammatory cytokine; signaling via its receptor (IL6R) is believed to contribute to development of inflammatory bowel diseases (IBD). The single nucleotide polymorphism rs2228145 in *IL6R* associates with increased levels of soluble IL6R (s-IL6R), as well as reduced IL6R signaling and risk of inflammatory disorders; its effects are similar to those of a therapeutic monoclonal antibody that blocks IL6R signaling. We used the effect of rs2228145 on s-IL6R level as an indirect marker to investigate whether reduced IL6R signaling associates with risk of ulcerative colitis (UC) or Crohn’s disease (CD). In a genome-wide meta-analysis of 20,550 patients with CD, 17,647 patients with UC, and more than 40,000 individuals without IBD (controls), we found that rs2228145 (scaled to a 2-fold increase in s-IL6R) was associated with reduced risk of CD (odds ratio 0.876; 95% confidence interval 0.822–0.933; *P* = .00003) or UC (odds ratio 0.932; 95% confidence interval 0.875–0.996; *P* = .036). These findings indicate that therapeutics designed to block IL6R signaling might be effective in treatment of IBD.

**What You Need to Know**

**Background and Context**

Interleukin-6 is a key cytokine in the pathogenesis of multiple inflammatory diseases. Whether interleukin-6 receptor (IL6R) blockade reduces the risk of developing inflammatory bowel disease (IBD) is unknown.

**New Findings**

The IL6R SNP rs2228145 has similar effects to pharmacological IL6R blockade and seems to protect individuals within a population from developing IBD.

**Limitations**

Despite evidence that IL6R blockade may prevent IBD in a healthy population, further studies are required to definitively infer that IL6R blockade prevents disease progression. Research into potential rare complications (e.g. perforations) is required.

**Impact**

Genetic evidence in humans supports IL6R signaling pathway as a drug target for IBD.

Crohn’s disease (CD) and ulcerative colitis (UC) are chronic inflammatory diseases affecting the gastrointestinal tract. Effective novel therapeutics remain a priority for these disabling conditions.

Interleukin-6 (IL6) is a proinflammatory cytokine that can exert its biological effect via 2 mechanisms: classic signaling through its membrane-bound IL6 receptor (IL6R), and trans-signaling, by binding to a soluble form of IL6R (s-IL6R) and subsequently to the membrane-bound transducer glycoprotein 130 (gp130).^1^ Whether IL6R is an attractive drug target for the management of both UC and CD is unknown.

Mendelian randomization (MR) can provide information on causality between an exposure and disease and has been successfully adopted for drug target validation.^2^ This method relies on a simple principle; if a modifiable exposure (eg, a biomarker, a complex trait, or an environmental risk factor, such as alcohol intake) is causal for a disease, then the genetic variants associated with (or that mirror the biological effects of) that exposure also will be associated with disease risk (Figure 1). The causal inference is possible due to the fundamental nature of the genome; variants are randomly allocated at meiosis, balancing confounders, and reverse causation, another important source of bias in observational studies, is not possible because the sequence of the germline is generally not modifiable by disease. MR can therefore be considered in many ways analogous to a randomized controlled trial (RCT) (Figure 2).

**Figure 1:**
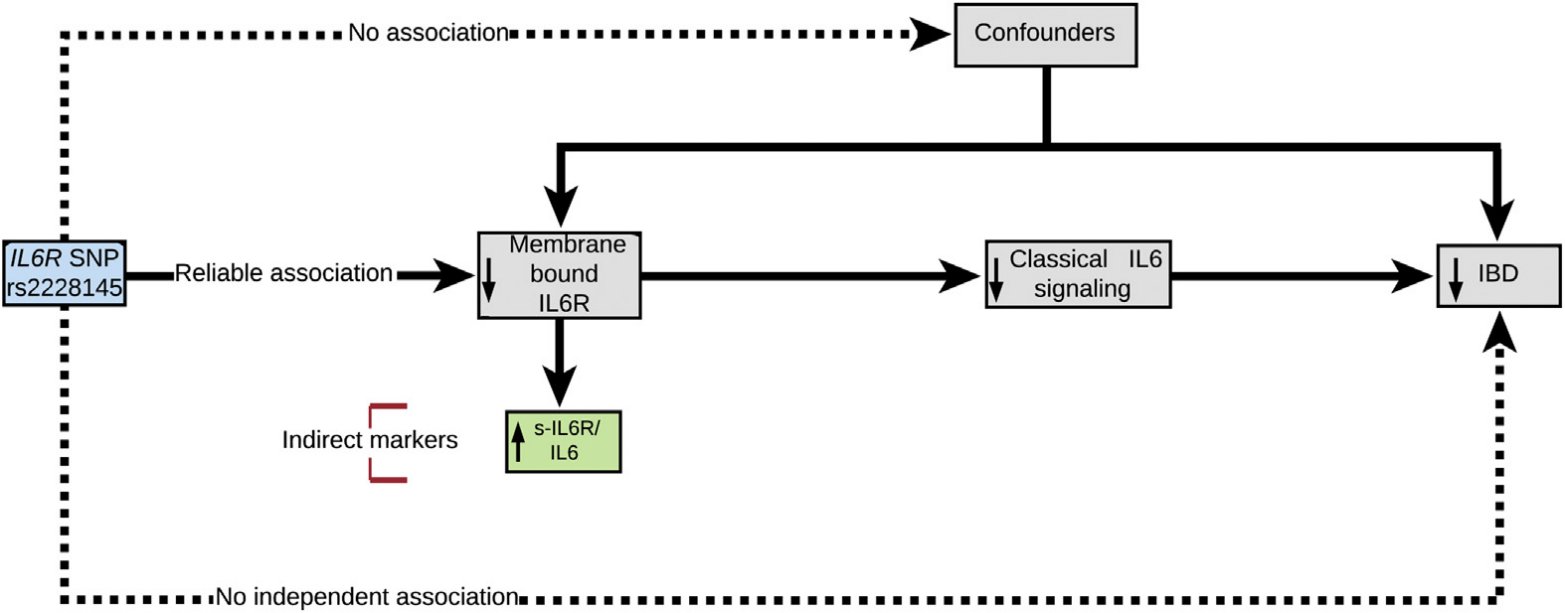
The MR model using a variant that disrupts normal function of the exposure (IL6R classical signaling), indirectly measured through increased levels of s-IL6R and IL6. The following are the 3 principles of MR analysis: a genetic instrument is robustly associated with the exposure (assumption 1, continuous arrow) but not with confounders (assumption 2, dotted arrow). The genetic variant is associated with the disease only through its effects on the exposure (assumption 3, dotted arrow).

**Figure 2:**
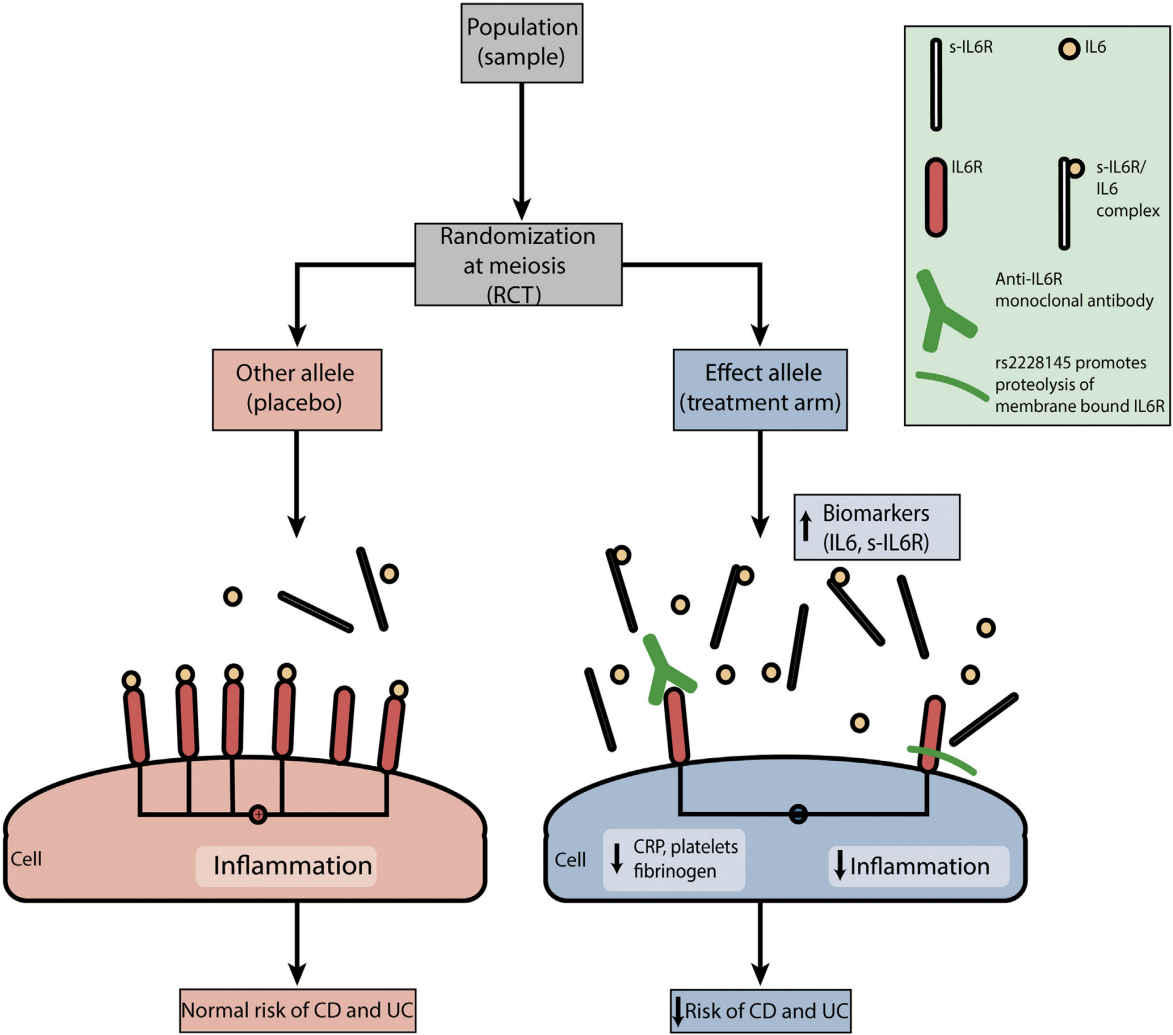
Schematic demonstrating how MR can be considered a natural analogue of the classic RCT (corresponding RCT steps in brackets). *IL6R* SNP rs2228145 has similar directional biomarker effects with tocilizumab. CRP, C-reactive protein.

The *IL6R* single nucleotide polymorphism (SNP) rs2228145 leads to increased proteolysis of its product and a reduction in classic signaling, and has similar directional effects to an existing anti-IL6R monoclonal antibody, tocilizumab (licensed for the treatment of rheumatoid arthritis); these include accumulation of circulating IL6 and s-IL6R levels, most likely due to reduced clearance of IL6 via its receptor in the liver and termination of negative feedback mechanisms, as well as a reduction in downstream inflammatory biomarkers, such as C-reactive protein, platelets, and fibrinogen.^3^ Despite important differences between tocilizumab and rs2228145 (the former inhibits both IL6R and s-IL6R, whereas the latter induces proteolysis specific to IL6R), the previously mentioned similarities make rs2228145 an attractive genetic instrument for drug target validation of IL6R inhibition (Figure 2).^4^ Previous MR studies have demonstrated protective associations between rs2228145 and inflammatory conditions, including coronary heart disease and rheumatoid arthritis.^3^

We aimed to evaluate and quantify the effect of IL6R signaling on the risk of developing UC and CD using a 2-sample MR design (see Supplementary Materials and Methods). We used the effect of rs2228145 on circulating levels of s-IL6R (and IL6, sensitivity analysis, see Supplementary Material) as an indirect marker for our exposure, reduced membrane-bound IL6R, and classic IL6R signaling, as described elsewhere (Figure 1).^2^, ^5^

The SNP (rs2228145) biomarker (s-IL6R, IL6) associations estimated in 1650 individuals were used as genetic instruments; rs2228145 elevates serum s-IL6R levels by 34% (IL6 by 15%).^4^ SNP–inflammatory bowel disease (IBD) associations were extracted from the largest IBD Genetics Consortium meta-analysis to date.^6^ The ratio MR method was used to obtain individual exposure estimates by dividing the SNP-outcome by the SNP-biomarker effect estimates.

In a combined total of 20,550 patients with CD (41,642 controls) and 17,647 patients with UC (47,179 controls), rs2228145 was associated with decreased odds of CD (odds ratio [OR] 0.948; 95% confidence interval [CI] 0.925–0.972; *P* = .00003) and UC (OR 0.973; 95% CI 0.948–0.998; *P* = .038) per effect allele. When applying the ratio MR method to quantify the association between the indirect marker (s-IL6R) and the outcome, a 2-fold genetic elevation of s-IL6R was associated with decreased odds of CD (OR 0.876; 95% CI 0.822–0.933; *P* = .00003) and UC (OR 0.932; 95% CI 0.875–0.996; *P* = .036) (see Supplementary Material). As a point of reference, tocilizumab increases s-IL6R levels by approximately 10-fold.^2^

Approximately 90% of drugs that enter clinical development fail; genetic evidence for a therapeutic target doubles the clinical success rate of such drugs.^7^ Our findings are consistent with 2 RCTs of antibodies targeting IL6 signaling for the treatment of CD; a small Phase I study of tocilizumab suggested higher clinical response rates in CD than the placebo group.^8^ Additionally, a recent RCT (ANDANTE) of an anti-IL6 antibody (PF-04236921) yielded higher clinical response and remission rates in patients with refractory CD vs placebo.^9^ Rare cases of gastrointestinal perforation, however, in patients treated with both of these antibodies remain a concern, because IL6 signaling may also contribute to epithelial repair of the intestinal mucosa.^10^ Avoiding use in at-risk individuals, including patients with diverticulitis and active fistulae, should still be advocated.

In the MR paradigm, genetic associations are generally free from confounding; previous studies have demonstrated no association between rs2228145 and age, birth weight, and education.^11^ Another concern for potential bias occurs when variants influence biomarkers on distinct causal pathways (horizontal pleiotropy). Genetic instruments, however, designed to model a protein (compared with more distal exposures such as complex traits) are protected from such pleiotropy.^12^ *IL6R* affects multiple downstream biomarkers on the same causal pathway, in a manner similar to pharmacological blockade, a (vertical) form of pleiotropy that does not lead to bias (Figure 2).

Interpretation of our results requires caution. A reduction in the risk of developing IBD does not necessarily translate to a reduction in disease progression, because genetic contributions to prognosis may differ from those of susceptibility.^13^ The nominal association (*P* = .036) with UC requires further validation. An incorrect conclusion would be that “increased s-IL6R reduces the risk of developing IBD”; s-IL6R was used as an indirect marker for reduced membrane-bound IL6R and subsequent classic signaling. Results should be interpreted as “a genetic reduction of IL6R and its classic signaling, sufficient to double s-IL6R, reduces the risk of developing IBD.”^2^

Our study suggests that a reduction in IL6R and subsequent classic signaling reduces IBD risk. One possible consequence is an increase in s-IL6R/IL6 complexes and subsequent trans-signaling via membrane-bound gp130. However, even if this occurs, it does not fully compensate for the impaired classic signaling, as evidenced by the net reduction in downstream biomarkers such as C-reactive protein, platelets, and fibrinogen. Alternatively, it has been proposed that the increase in s-IL6R may actually further reduce trans-signaling by enhancing the buffering potential of the abundant soluble glycoprotein 130.^14^ Further work is required to determine the effect of rs2228145 on IL6 trans-signaling and whether trans-signaling is in itself a potential therapeutic target in IBD.

On the basis of genetic evidence in humans, IL6R signaling seems to have a causal role in the development of both CD and UC. Suitably powered RCTs of new and existing therapeutics targeting this complex pathway are required in both conditions, alongside ongoing focus on the pathophysiology underlying rare complications, such as gastrointestinal perforations.

## Figures and Tables

**Supplementary Table 1:** The SNP–s-IL6R and SNP-IL6 Associations (per Effect Allele) Generated From a Previous Study Were Used as Genetic Instruments in This Report

Gene	SNP	EA	OA	Biomarker	ln(levels)	SE
IL6R	rs2228145	C	A	s-IL6R	0.2949	0.0148
IL6	rs2228145	C	A	IL6	0.1362	0.0176

CRP, C-reactive protein; EA, effect allele; OA, other allele; SE, standard error.

**Supplementary Table 2:** SNP-CD and SNP-UC (per Effect Allele) Associations Downloaded From Available Genome-Wide Association Study Summary Statistics, Presented Here After Data Harmonization

Study	Disease	SNP	EA	OA	ln(levels)	SE
Liu^6^	CD	rs2228145	C	A	−0.0530323	0.0126631
Liu^6^	UC	rs2228145	C	A	−0.0276154	0.0131298

CRP, C-reactive protein; EA, effect allele; OA, other allele; SE, standard error.

## Supplementary Materials and Methods

### Mendelian Randomization

MR requires that the genetic instrument is associated with a modifiable exposure of interest, and any association between the instrument and the outcome is mediated by the exposure through its downstream effects. MR assesses the causal effect of an exposure (in this article, we use increased s-IL6R level as an indirect measure for our exposure, reduced IL6R and subsequent impaired IL6R signaling, as per previous studies^1^, ^2^) on an outcome (eg, IBD) by grouping individuals in the population according to the possession of the genetic variants that modify an exposure of interest; genetic variants should be free from conventional confounding because they are “assigned” randomly at meiosis; reverse causality bias is impossible because the germline genome is generally nonmodifiable by disease. MR can be thought of as analogous to an RCT that uses genetic variation as the method of randomization; the key difference is that MR can be performed at any time using routine genotype data, without the exposure of patients to the drug. Advances on MR methodology have allowed for the measurement of genetic effects on exposure and outcomes in independent samples (2-sample MR); this has enabled the use of publically available results from very large genome-wide association studies for both risk factor “exposures” and disease “outcomes” to evaluate causal relationships.^3^ By using genetic variants that effect known drug targets and have quantifiable effects, MR offers a relatively unbiased approach for the prediction of both intended (eg, RCT endpoints) and unintended (eg, drug repurposing opportunities, adverse events) drug effects.

### Genetic Datasets Used

SNP-CD, SNP-UC, ln (OR) and standard errors were downloaded from the most recent IBD Genetics Consortium meta-analysis (https://www.ibdgenetics.org/downloads.html).

### Study Outcome

The primary outcome was the risk of developing (1) CD (2) UC per 2-fold elevation in circulating s-IL6R as an indirect measure for our exposure (genetically impaired IL6R signaling).

### Statistical Analysis

The SNP-IBD associations were extracted as described previously and presented per effect allele. The SNP–s-IL6Rs were estimated in 1650 individuals included in a large collaborative MR study.^4^ These estimates were presented in percentage differences, which were converted to ln-transformed units.

SNP–s-IL6R and IL6 associations were collected in ln-transformed units from previous publications (Supplementary Table 1).

The ratio MR method was used. This method was used to obtain individual SNP estimates by dividing the SNP – outcome (Supplementary Table 2) by the SNP – biomarker effect estimates. Analyses were performed using R package MR-Base, version 3.2.4 (http://www.r-project.org).^5^

OR estimates of UC and CD were transformed (for ease of interpretation). Results were presented per 2-fold increase in circulating s-IL6R and IL6 levels:

$${\left(\sqrt[{e}]{OR}\right)}^{2}\text{,}$$

where OR is the odds ratio estimate per 1-ln increment in biomarker levels and *e* is the base of the natural logarithm.

### Sensitivity Analysis

When applying the ratio MR method to model the association between the indirect marker IL6 and the outcome, a 2-fold genetic elevation of IL6 was associated with decreased odds of CD (OR 0.75; 95% CI 0.81–0.94; *P* = .00003) and UC (OR 0.861; 95% CI 0.71–1; *P* = .038).
